# Identification and phylogenetic analysis of RNA binding domain abundant in apicomplexans or RAP proteins

**DOI:** 10.1099/mgen.0.000541

**Published:** 2021-03-03

**Authors:** Thomas Hollin, Lukasz Jaroszewski, Jason E. Stajich, Adam Godzik, Karine G. Le Roch

**Affiliations:** ^1^​ Department of Molecular, Cell and Systems Biology, University of California Riverside, 900 University Avenue, Riverside, CA 92521, USA; ^2^​ Department of Biomedical Sciences, University of California Riverside School of Medicine, 900 University Avenue, Riverside, CA 92521, USA; ^3^​ Department of Microbiology and Plant Pathology, Institute for Integrative Genome Biology, University of California Riverside, 900 University Avenue, Riverside, CA 92521, USA

**Keywords:** phylogenetic tree, protein structure, RAP domain, RNA-binding protein

## Abstract

The RNA binding domain abundant in apicomplexans (RAP) is a protein domain identified in a diverse group of proteins, called RAP proteins, many of which have been shown to be involved in RNA binding. To understand the expansion and potential function of the RAP proteins, we conducted a hidden Markov model based screen among the proteomes of 54 eukaryotes, 17 bacteria and 12 archaea. We demonstrated that the domain is present in closely and distantly related organisms with particular expansions in Alveolata and Chlorophyta, and are not unique to Apicomplexa as previously believed. All RAP proteins identified can be decomposed into two parts. In the N-terminal region, the presence of variable helical repeats seems to participate in the specific targeting of diverse RNAs, while the RAP domain is mostly identified in the C-terminal region and is highly conserved across the different phylogenetic groups studied. Several conserved residues defining the signature motif could be crucial to ensure the function(s) of the RAP proteins. Modelling of RAP domains in apicomplexan parasites confirmed an ⍺/β structure of a restriction endonuclease-like fold. The phylogenetic trees generated from multiple alignment of RAP domains and full-length proteins from various distantly related eukaryotes indicated a complex evolutionary history of this family. We further discuss these results to assess the potential function of this protein family in apicomplexan parasites.

## Data Summary

Table S1 (available with the online version of this article) shows the full list of proteomes used and their corresponding accession numbers.

**Impact Statement**

In eukaryotes, post-transcriptional regulation of gene expression requires various mechanisms and involves many RNA-binding proteins essential to RNA biology at multiple levels, including splicing, stability, localization and translation. Here, we demonstrate that the ‘RNA binding domain abundant in apicomplexans’, or RAP family, is expanded not only in Apicomplexa but also in Alveolata and Chlorophyta. Phylogenetic and structural analysis of the RAP proteins showed a conservation of the RAP domain, while the N-terminal regions are divergent. These RAP proteins seem to be essential for *Plasmodium falciparum* and *Toxoplasma gondii*, two major apicomplexan parasites that remain of global public-health concern. This study opens up new perspectives to understand this poorly characterized protein family and explore its potential as novel therapeutic targets to counter the threat of drug resistances.

## Introduction

Several severe human diseases are caused by protozoans, such as malaria by *Plasmodium* species or toxoplasmosis by *Toxoplasma gondii*. These parasites are members of the phylum Apicomplexa, belonging to the Stramenopile, Alveolate and Rhizaria (SAR) supergroup, which possess a plastid called an apicoplast and an apical secretory system composed of micronemes, rhoptries and dense granules. Despite a large decrease in global malaria deaths in the last two decades, 405 000 persons succumbed to this disease in 2018, indicating that it is still a major public-health concern [1]. In addition, the emergence of drug resistance, against all antimalarials used today, amplifies the importance of discovering new therapeutic targets against this devastating disease.

**Fig. 1.**
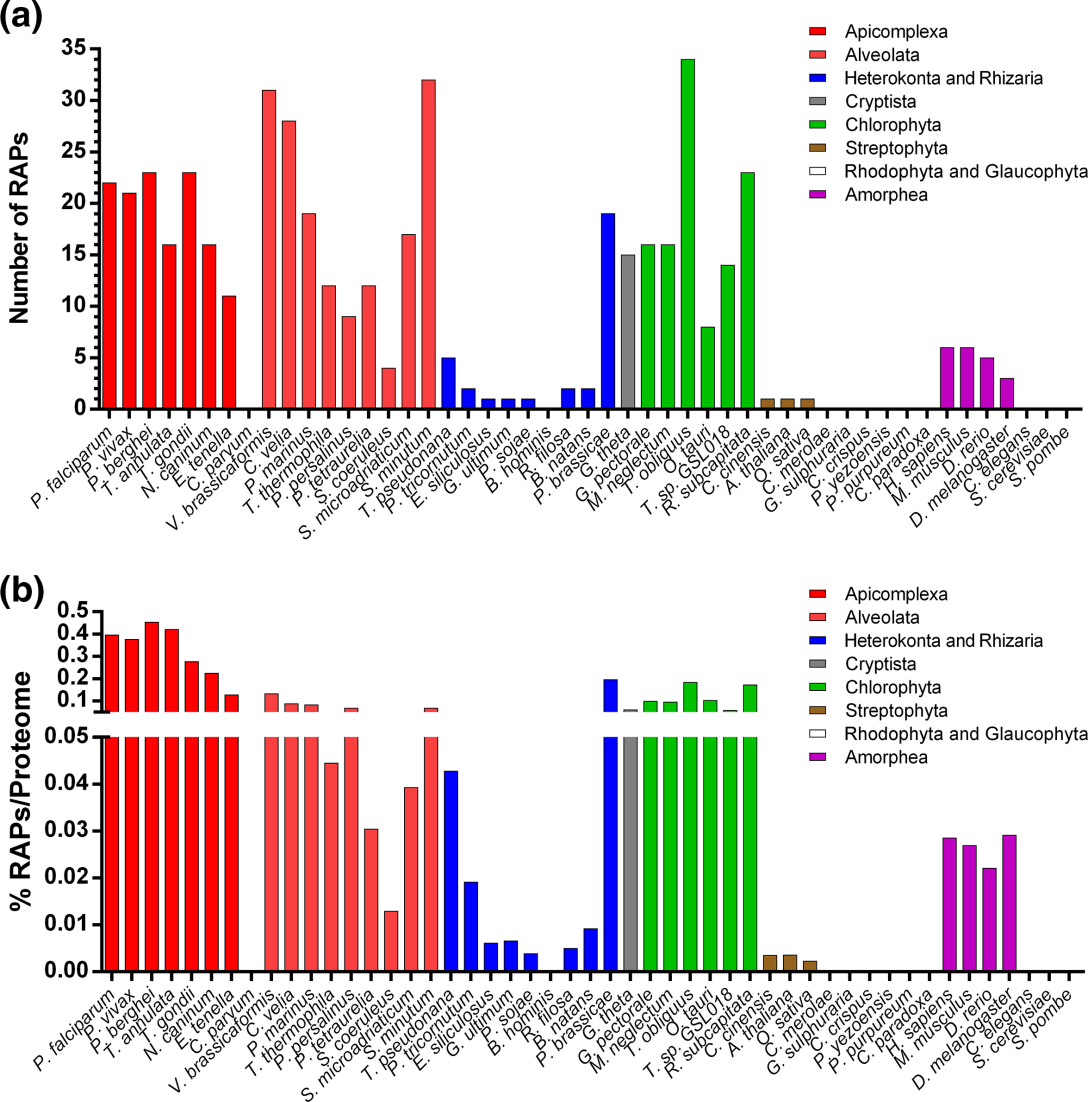
Frequency of RAP proteins in different eukaryotic organisms. (a) The total number of RAP proteins discovered by HMM for each species. (b) Percentage of RAP proteins identified and normalized by the size of the proteome. Some species are excluded to simplify the visualization.

**Fig. 2.**
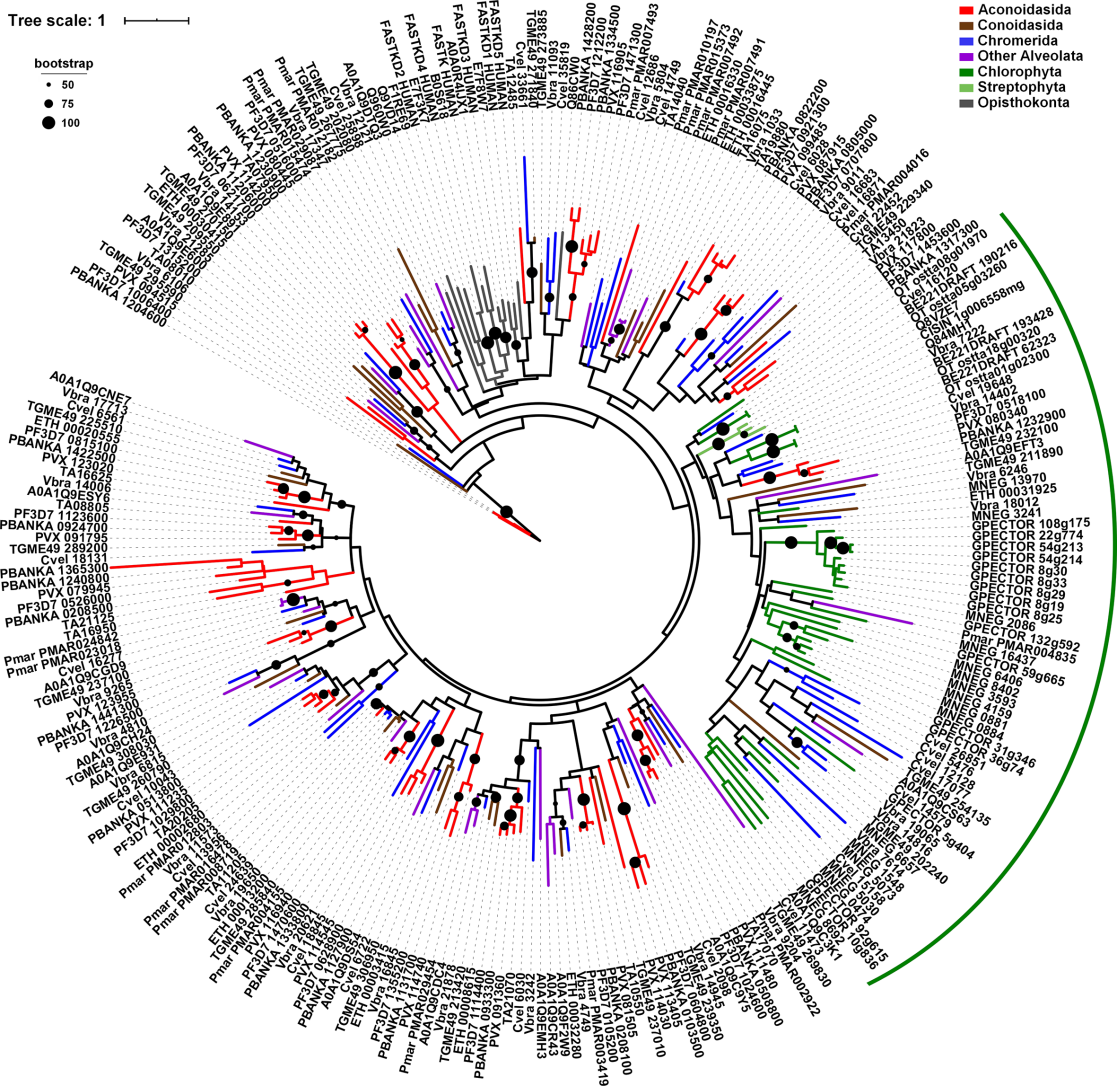
Phylogenetic analysis of the RAP domain in Eukaryota. The maximum-likelihood tree is built from an alignment of RAP domains extracted from 267 proteins corresponding to 19 different species. Alveolates are represented by Aconoidasida (*Plasmodium falciparum*, *Plasmodium vivax*, *Plasmodium berghei* and *Theileria annulata*) in red, Conoidasida (*Toxoplasma gondii* and *E. tenella*) in brown, Chromerida (*V. brassicaformis* and *Chromera velia*) in blue, and *Perkinsus marinus* and *Symbiodinium microadriaticum* in purple. Viridiplantae are represented by Chlorophyta (*G. pectorale*, *Monoraphidium neglectum* and *Ostreococcus tauri*) in green, and Streptophyta (*A. thaliana*, *Oryza sativa* and *Citrus sinensis*) in pale green. Opisthokonta (*H. sapiens*, *Danio rerio* and *Drosophila melanogaster*) are indicated in grey. The green arc indicates the clade enriched in RAP proteins of Chlorophyta species. Bootstrap values (>50 %) are shown on respective branches. The scale indicates the number of substitutions per site.

**Fig. 3.**
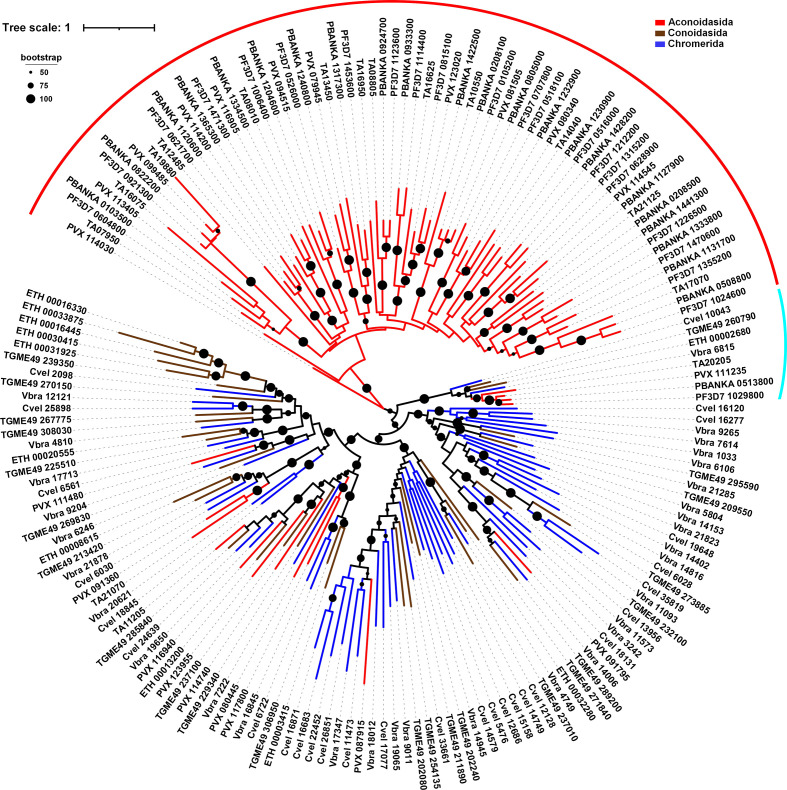
Phylogenetic analysis of full-length RAP proteins in Apicomplexa and Chromerida. The maximum-likelihood tree is built from 175 protein sequences corresponding to eight different species. Aconoidasida (*Plasmodium falciparum*, *Plasmodium vivax*, *Plasmodium berghei* and *Theileria annulata*) are represented in red, Conoidasida (*Toxoplasma gondii* and *E. tenella*) in brown, and Chromerida (*V. brassicaformis* and *Chromera velia*) in blue. The red arc shows the clade formed by the Aconoidasida. The turquoise arc indicates the RAP protein conserved in different species. Bootstrap values (>50 %) are shown on respective branches.The scale indicates the number of substitutions per site.

To systematically identify essential genes, large-scale genetic screening methodologies using *piggyBac* transposon or CRISPR–Cas9 technologies have been recently developed in *Plasmodium falciparum* and *Toxoplasma gondii* [2, 3]. Several enriched or parasite-specific protein domains were identified as essential in these initial screens, suggesting that these protein families may be good candidates for novel drug targets [4]. One such domain identified was the ‘RNA binding domain abundant in apicomplexans’ or RAP domain. The RAP-containing genes are enriched in Apicomplexa with at least 11–20 members in each of these genomes, while mammalian genomes typically have around 6 [5]. In the following text, we refer to proteins containing RAP domains simply as ‘RAP proteins’. The enrichment of RAP proteins is extreme in apicomplexan parasite genomes as they encode only 4000–7000 genes compared to ~20 000–30 000 genes in mammals. The essentiality of the RAP protein family and its expansion in Apicomplexa could offer an opportunity to selectively target these proteins with reduced probability of causing host toxicity.

Despite the potential of these proteins as novel drug targets, the functions of RAP proteins are still poorly characterized. RAP proteins are predicted to be involved in RNA binding [5] and likely have a function in the mitochondria [6, 7] or the chloroplasts [8–10]. Studies in model organisms have confirmed a role for RAP proteins in RNA metabolism. In the green alga *Chlamydomonas reinhardtii*, the RAP proteins Raa3 and TDA1 contribute, respectively, to the trans-splicing of the chloroplast mRNA *psaA* [8] and to trap *atpA* transcripts and activate their translation [9]. In humans, six RAP proteins have been identified and annotated as FASTK (Fas activated serine*/*threonine kinase) for the initially proposed role of the first characterized member of this family to phosphorylate TIA-1, an effector of apoptosis [11]. Later, the RAP domain of FASTK was demonstrated as being essential in regulation of mitochondrial ND6 mRNA levels [7] and disruption of FASTK domain 1 (FASTKD1) by CRISPR–Cas9 led to an increase in ND3 transcripts, suggesting a role in RNA stability [12]. Similar data from the knock-out of FASTKD4 confirmed its involvement in mitochondrial mRNA processing and/or stabilization [12]. Knock-out of FASTKD3 demonstrated a decrease in translation efficiency of COX1 leading to a reduction of the mitochondrial complex IV activity [13], while an assembly defect of the complex was observed when FASTKD5 was silenced by siRNA (small interfering RNA) [14]. Finally, siRNA of FASTKD2 led to a decrease in the ribosomal subunit causing alteration of the oxidative phosphorylation system assembly [14]. These RAP proteins also have two domains annotated FAST_1 and FAST_2 whose function remain unknown. A review depicting the role of these different proteins in more details has been recently published by Jourdain and colleagues [15].

The protein domain architecture of RAP proteins typically consists of two different regions, the RAP domain and a helical N-terminal region of variable length. The most conserved part of the RAP domain contains approximately 60 amino acids including multiple aromatic and charged residues [5]. No experimental crystal structure of the RAP domain has yet been solved; however, prediction algorithms suggest that the RAP domain may have adapted a restriction endonuclease-like fold with α/β-sandwich topology [5, 12]. Previous studies reported that mutations in the N-terminal region led to an RNA mistargeting, suggesting its importance for the function of the RAP proteins [12, 16]. In some RAP proteins, from *Plasmodium* to human, the N-terminal domain consists of helical repeat protein motifs, similar to those identified in tetra- (TPR), penta- (PPR), hepta- (HPR) and octotricopeptide repeat (OPR) proteins [17–22]. These proteins contain repeats of ~34–40 amino acids and typically form superhelical scaffolds mediating protein–protein or RNA–proteins interactions for TPR and PPR/HPR/OPR proteins, respectively [23–26]. PPR/HPR/OPR proteins are highly associated with mitochondria and chloroplasts, and have been reported to play an essential role in RNA and translation regulation in these particular organelles in various organisms, including green algae, plants and *Plasmodium* [20, 21, 26–32]. The presence of these peptide repeats in some RAP proteins from different species led to their annotation as HPR or OPR proteins.

Despite these observations, the function of the RAP domain in apicomplexan parasites remains obscure. Here, we performed a global analysis of RAP protein distribution and found that the expansion of this domain was not unique to Apicomplexa, but also includes Alveolata and Chlorophyta. Phylogenetic analysis of the RAP domain showed its conservation, while the N-terminal regions are very divergent, especially in Aconoidasida. Our study provides a global overview of this poorly characterized protein family and raises questions about expansions of RAP proteins in organisms possessing mitochondrion and plastid, and on their roles in the function or communication of these two organelles.

## Methods

### Hidden Markov models (HMMs)

Proteomes were obtained from different databases as reported in Table S1, corresponding to 54 eukaryotes, 17 bacteria and 12 archaea. Each proteome was scanned for RAP domains using Pfam domain PF08373.10 and hmmscan 3.3 (http://hmmer.org/) [33] with the following settings: -E 0.000001 --domE 0.00001 F3 0.01. Extracted results are shown in Table S1 and additional RAP proteins undetected by the search are indicated.

### Phylogenetic trees

The full-length sequence of the proteins identified or a sequence of 100 amino acids including the RAP domain were extracted and aligned with Clustal Omega v1.2.4 [34] on EMBL-EBI [35]. Phylogenies were reconstructed using the maximum-likelihood criterion implemented in iq-tree v1.6.12 [36] and branch supports were inferred using 500 bootstrap replicates. The best-fit model was chosen according to the Bayesian information criterion, corresponded to VT+F+R5, VT+R4 and VT+R5, respectively. The different phylogenetic trees were visualized and edited with Interactive Tree Of Life (iTOL) v5 (https://itol.embl.de/) [37].

### Domain and motif discovery

To identify protein domains present in RAP proteins, we performed a smart analysis with default settings and Pfam domains [38]. The analysis included the full-length sequence of 267 RAP proteins from *Plasmodium falciparum*, *Plasmodium vivax*, *Plasmodium berghei*, *Theileria annulata*, *Toxoplasma gondii*, *Eimeria tenella*, *Vitrella brassicaformis*, *Chromera velia*, *Gonium pectorale*, *Monoraphidium neglectum*, *Ostreococcus tauri*, *Homo sapiens*, *Danio rerio* and *Drosophila melanogaster*. The results are detailed in Table S1.

To identify conserved motifs, the MEME Suite v5.1.0 [39] was used on the full-length sequence of RAP proteins from three different groups. The Apicomplexa–Chromerida group contains *Plasmodium falciparum*, *Plasmodium vivax*, *Plasmodium berghei*, *Theileria annulata*, *Toxoplasma gondii*, *E. tenella*, *V. brassicaformis* and *Chromera velia*. The Chlorophyta group is composed of *G. pectorale*, *Monoraphidium neglectum* and *Ostreococcus tauri*, while *H. sapiens*, *Mus musculus*, *Danio rerio* and *Drosophila melanogaster* form the Metazoa group. A randomized group was composed with 20 random sequences from each group. The total number of motifs was set up at five and the width from 6 to 100 amino acids.

### Analysis of the RAP repertoire in *Plasmodium falciparum*, *Plasmodium berghei* and *Toxoplasma gondii*

The protein sequences of the respective RAPs from *Plasmodium falciparum*, *Plasmodium berghei* and *Toxoplasma gondii* were extracted from PlasmoDB v46 and ToxoDB v46. The homologues of *Plasmodium falciparum* in *Plasmodium berghei* were obtained by PlasmoDB. For *Toxoplasma gondii*, a blast search was performed on *Plasmodium falciparum* 3D7 proteome (PlasmoDB) for each RAP protein and the best result was reported. The percentage of identity between protein sequences has been determined by Needleman–Wunsch global alignment. To predict the subcellular localization, TargetP 1.1, MitoFates and MitoProt II were used for potential localization in the mitochondrion [40–42], and PlasmoAP and pats for potential localization in the apicoplast [43, 44]. All prediction tools were used with their default settings.

### Structure predictions

Optimal modelling template and boundaries of structural RAP domains in *Plasmodium* proteins were identified using the HHpred server [45] by searching the database of HMMs representing protein chains from the PDB database (PDB_mmCIF_23_Jul) with sequences of *Plasmodium* RAP proteins (Table S1). The sequence of one of the two top hits (6rd6 chain 2 from *Polytomella* sp*.* Pringsheim 198.80) was then used in turn as a query to search HMMs representing *Plasmodium* proteins (Euk_Plasmo dium_falciparum_3d7_7_ Jun_2017) to check which of the *Plasmodium* RAP proteins can be fully aligned with both helical repeats and ⍺/β domains of 6rd6 chain 2. Out of 22 RAP proteins listed in Table S1, 17 aligned with both domains and were included in the subsequent analysis.

In the next step, the HHpred alignment of the ⍺/β domain of 6rd6 chain 2 with these 17 proteins was used to identify long asparagine-rich inserts in their RAP domains (these were asparagine-rich regions longer than 50 and not aligned with the ⍺/β domain of 6rd6 chain 2 residues 322–445). Such regions were identified in 3 out of 17 RAP domains, and they were removed to shorten and stabilize the alignment (their positions are marked by dark vertical bars) Fig. 5.

The final alignment of RAP domains from 17 *Plasmodium* proteins and the modelling templates was then calculated with muscle [46]. Superpositions of protein structures were calculated with fatcat [47] and posa [48]. Structural models were obtained with Modeller [49]. The alignment was rendered with ENDscript [50] and protein structures were rendered with PyMOL [51]

## Results

### RAP proteins across various and distant organisms

To explore the evolution of the RAP proteins, we first used a HMM of the RAP domain from Pfam (PF08373.10) to search the proteomes of 54 eukaryotes, 17 bacteria and 12 archaea [52]. These organisms were selected to cover a broad phylogenetic span with particular attention to the SAR supergroup. Our HMM scan identified 463 RAP domain copies in the 83 species. To obtain a broader and more accurate view of the RAP protein distribution, this analysis was compared to previously published datasets that identified RAP proteins in some of the selected organisms [53–55]. These datasets were merged with the results of our search and yielded in a total of 487 predicted RAP proteins (Fig. 1a, Table S1), of which 24 RAPs (4.9 %) were not detected by our HMM search and were added manually. Our HMM analysis identified 103 new potential RAPs, including 35 from three recently sequenced genomes (*Reticulomyxa filosa*, *Bigelowiella natans* and *Symbiodinium minutum*). We do note that the variability in quality of genome assemblies and gene predictions can impact the number of identified putative RAP proteins for some organisms.

Our results confirmed previous observations that RAP proteins are abundant in apicomplexan parasites [5, 54]. In *Toxoplasma* and *Plasmodium* species, between 21 to 23 RAP proteins were identified corresponding to about 0.4 % of the proteome (Fig. 1a, b). A high number of RAP proteins were observed in *Theileria* and *Neospora* (16 RAPs) and in *Eimeria* (11 RAPs). Similar to a previous report [53], no RAP proteins were found in *Cryptosporidium parvum*, an apicomplexan parasite that has lost its apicoplast and possess a mitochondrion-derived compartment [56, 57].

A large number of RAP proteins were observed within the Chromerida, which are unicellular photosynthetic organisms that belong to the superphylum of Alveolata and are closely related to Apicomplexa. The species *V. brassicaformis* and *Chromera velia* have 31 and 28 predicted RAP proteins, respectively (~0.1 % of proteome), comparable to a previous report [53]. Other alveolates also have a large number of domains including *Perkinsus marinus* with 19 RAP proteins and *Symbiodinium,* a family of endosymbiotic dinoflagellates that have between 17 to 32 RAP proteins identified in their genomes. For the Ciliophora, a group of protozoans characterized by the presence of hair-like organelles, the distribution seems to be more variable with 4 RAP proteins for *Stentor coeruleus* and 11 to 12 for *Paramecium tetraurelia*, *Pseudocohnilembus persalinus* and *Tetrahymena thermophila* (0.01–0.06 % of proteome). Among other members of the SAR supergroup, the Heterokonta, algae ranging from the giant multicellular kelp to the unicellular diatoms, and the Rhizaria, mostly unicellular eukaryotes that are non-photosynthetic with a symbiotic relationship with unicellular algae, the number of RAP proteins detected is quite low (0 to 6 RAPs, 0–0.04 % of proteome). One exception is *Plasmodiophora brassicae*, a parasite that is responsible for the club root disease of crucifers and which possesses 19 RAP proteins (~0.2 % of proteome). Altogether, the results showed that the Alveolata protists possess a large repertoire of RAP proteins.

Interestingly, in the Archaeplastida, a major group of autotrophic eukaryotes that include red algae, green algae and land plants, an irregular distribution of RAP proteins was observed. No RAP proteins were detected among five Rhodophyta and one Glaucophyta selected. Among Viridiplantae, we noticed only one RAP among three Streptophyta (~0.003 % of proteome), whereas in six Chlorophyta species the median number of RAP proteins is 16 (~0.1 % of proteomes), indicating an expansion of this domain after the lineage diverged.

Within the human genome, we detected six RAP proteins, similar to prior results [5, 15]. Generally, there are around 5–6 RAP proteins in the Chordata (~0.03 % of proteome), whereas only 3 RAP proteins were identified in *Drosophila*. The RAP domain seems to be absent, or at least undetectable using our computational pipeline, in other Opisthokonta and relatives including the Amoebozoa, Nematoda, Ascomycota (Fungi) and Excavata. Nevertheless, while we were not able to detect any RAP proteins in our selected nematode, *Caenorhabditis elegans*, a single RAP protein was identified by the Pfam database in the genome of the nematodes *Strongyloides papillosus*, *Strongyloides venezuelensis* and *Parastrongyloides trichosuri*, suggesting this phylum may not be completely depleted of RAP proteins. Such data indicate that the distribution of the RAP domain is irregular and could be explained by multiple losses of the RAP-containing genes across lineages.

Finally, although the majority of the 12 archaeal and 17 bacterial proteomes that we analysed appear to lack a clear RAP domain, we were able to detect one to two RAP candidates in some of these organisms, including '* Methanomicrobia *' species (Table S1). Altogether, these observations suggest expansions of the RAP family may have occurred in the common ancestor of the Alveolata and in Chlorophyta, and indicates this domain is not uniquely abundant in apicomplexan parasites.

### Phylogenetic analysis of RAP domains

To examine further the evolution of these RAP proteins, we isolated a region of 100 amino acids including the RAP domain from 267 sequences of six apicomplexan parasites (*Plasmodium falciparum*, *Plasmodium berghei*, *Plasmodium vivax*, *Theileria annulata*, *Toxoplasma gondii* and *E. tenella*), two Chromerida (*V. brassicaformis* and *Chromera velia*), two SAR supergroup members (*Perkinsus marinus* and *Symbiodinium microadriaticum*), three Chlorophyta (*G. pectorale*, *Monoraphidium neglectum* and *Ostreococcus tauri*), three Streptophyta (*Arabidopsis thaliana*, *Citrus sinensis* and *Oryza sativa*), and three Metazoa (*H. sapiens*, *Danio rerio* and *Drosophila melanogaster*). We found that among proteins larger than 200 amino acids, ~91 % of the RAP domains are located in the C-terminal of the proteins, indicating that this localization seems important for the RAP function (Fig. S1).

We then generated a phylogenetic tree by first aligning the RAP domains with Clustal Omega [34] and inferring a maximum-likelihood tree with iq-tree [36]. We were unable to detect any split into separate branches, demonstrating for the first time that RAP domains did not diverge early, suggesting that this domain is well conserved across all organisms selected (Fig. 2). The Chlorophyta RAP domains are distributed globally in a single clade, which could be due to the acquisition of certain specific characteristics of this phylogenetic group or to the expansion of this family from a single or a limited number of ancestors (green arc).

### Phylogenetic analysis of full-length RAP proteins

To further explore the evolution of the RAP proteins and distinguish between protein duplication that could undergo speciation or protein shuffling in some specific lineages, we inferred a phylogenetic tree based on the full-length protein alignment. We first analysed 175 protein sequences found in six apicomplexan parasites and two chromerids. The quality of the alignment obtained from these sequences was low. This can be explained by the large number of proteins, their variable length, and the absence of highly conserved regions besides helical repeats and the RAP domain. This was particularly true for the N-terminal part of the proteins. Considering the low quality of our alignments, it is entirely possible to speculate that the phylogenetic tree generated from the full-length proteins leads to some uncertainty. However, data extracted from the Aconoidasida, including the three *Plasmodium* species and *Theileria*, suggest that RAP proteins from these organisms form a clade (Fig. 3, red arc), whereas the remaining proteins form four clades each containing members of both Conoidasida (*Toxoplasma* and *Eimeria*) and Chromerida (Fig. 3). The atypical A/T richness of *Plasmodium falciparum* genome is insufficient to explain this grouping, since *Plasmodium vivax* and *Theileria annulata* exhibit a more classical A/T content (57–67 %) [58, 59]. In total, 66/82 RAP proteins (80 %) from these species are present in this part of the tree. Interestingly, only one RAP from *Plasmodium falciparum* and *Plasmodium berghei* do not belong to the clade and are grouped with one RAP from each selected species (Fig. 3, turquoise arc). This relationship is not unexpected, since PF3D7_1029800 shares 48 % of identity with TGME49_260790, whereas all others *Plasmodium falciparum* RAP proteins present low similarity with *Toxoplasma gondii* copies (Fig. S2, Table S2). Further analysis of these RAP proteins showed that residues are globally conserved, including in the peptide repeats and the RAP domain, with the exception of some insertions present in few species, while the second half of the repeat region presents greater variability (Fig. S2).

We then reconstructed a phylogenetic tree of RAP proteins from the complete set of species selected in this study (Fig. S3). As previously observed, Aconoidasida are separated (72/82) from the other species in the SAR supergroup (red arc). PF3D7_1029800 and its homologues form a monophyletic clade with one RAP protein from *Perkinsus marinus* and *Symbiodinium microadriaticum* (turquoise arc), confirming that this particular protein is conserved across all alveolates and was likely present in their common ancestor. This phylogenetic tree also revealed that around 56 % (22/39) of the examined RAP proteins from the green algae form a distinct clade. Interestingly, the higher plants and Metazoa are not grouped with green algae, suggesting this divergence appeared after the dissociation of this phylogenetic group. It is tempting to speculate that some RAP proteins may have separated early and form their own branches, while other may have duplicated recently and form an organism specific sub-branch. Taken together, these data suggest that the incongruence observed in the examined phylogenetic trees may not be due to the RAP domain but instead to the N-terminal region, which may facilitate the specificity of the protein in RNA targeting.

### Motif identification in RAP proteins

To verify the presence of other domains in RAP proteins, we performed a smart analysis [38] on the complete set of proteins selected in our study. As expected, our results illustrated that the RAP domain is the main motif identified (71 %), confirming the data extracted from our HMM search (Fig. S4). The lack of detection of the RAP domain in some of our proteins can be explained by the use of an alternative tool and filter stringency. This was indeed confirmed by the fact that several RAP proteins identified by Interpro were also not detected in this smart analysis (Table S1). However, despite the tools used, no other domain was detected as highly enriched in our selected RAP proteins. Only two motifs, FAST_1 and FAST_2, discovered in FASTK proteins [15], were detected in 6 and 4.5 %, respectively, of our selected candidates. As previously indicated, these two motifs seem to be specific to Metazoa [15]. However, FAST_1 was identified in two *Plasmodium* RAP proteins (PVX_114200 and PBANKA_1120600) and one in *G. pectorale* (A0A150GDR9). A few other domains, such as Mpp10, Aquarius_N and DEXDc, known to be involved in RNA biology, were also identified but none could be detected more than twice. These results indicate that apart from the RAP domain, and to a lesser extent the FAST domains, these proteins do not require other known domains to ensure their biological function.

To further investigate the conservation of the RAP domain and identify additional overrepresented motifs, we used the MEME Suite [39] on the full-length RAP proteins. The analysis was performed on three groups: Apicomplexa–Chromerida (annotated Api), Chlorophyta (Chl) and Metazoa (Met) as previously described with the addition of *Mus musculus* to increase Metazoa sampling. A total of five motifs were searched for each group. In Apicomplexa–Chromerida group (175 sequences), we only detected two distinct motifs, Api1 and Api2, highly conserved in the majority of the proteins, both corresponding to parts of the RAP domain (Fig. S5). Three other motifs, APi3, APi4 and APi5, were only detected in a few proteins. Api3 and Api5 partially overlap with the RAP domain, whereas Api4 was found specifically in the N-terminal region of the proteins. Similar results were obtained for the green algae (39 sequences), with Chl4 and Chl5 covering the RAP domain and Chl1 replacing Chl4/Ch5 in some proteins in *G. pectorale*. Chl2 and Chl3 were detected for approximately 20 % of the sequences, but do not seem to be conserved in most organisms with the exception of *G. pectoral*e. For the Metazoa (20 sequences), we only detected Met1 related to the RAP domain, while Met2/Met3 and Met4/Met5 were associated with the FAST_2 and FAST_1 domain, respectively. To validate the motifs that we identified across the different groups, we randomly selected 20 RAP proteins from each of the three groups (annotated Ran1–5) and ran the MEME Suite software. We found Ran1 and Ran2 motifs in the RAP domain with sequences similar to the previously defined motifs (Fig. S5). Ran4 and Ran5 were found only in metazoan sequences and matched Met2 and Met3, which correspond to the motifs identified in FAST_1. Interestingly, Ran3 was identified in 51 out of 60 sequences upstream of the RAP domain. Although the OPR-specific PPPEW sequence was not identified in this motif, close examination of Ran3 indicated that the degenerate consensus sequence was based on a mix between HPR and OPR motifs [21, 60], validating their presence in different phyla.

**Fig. 4.**
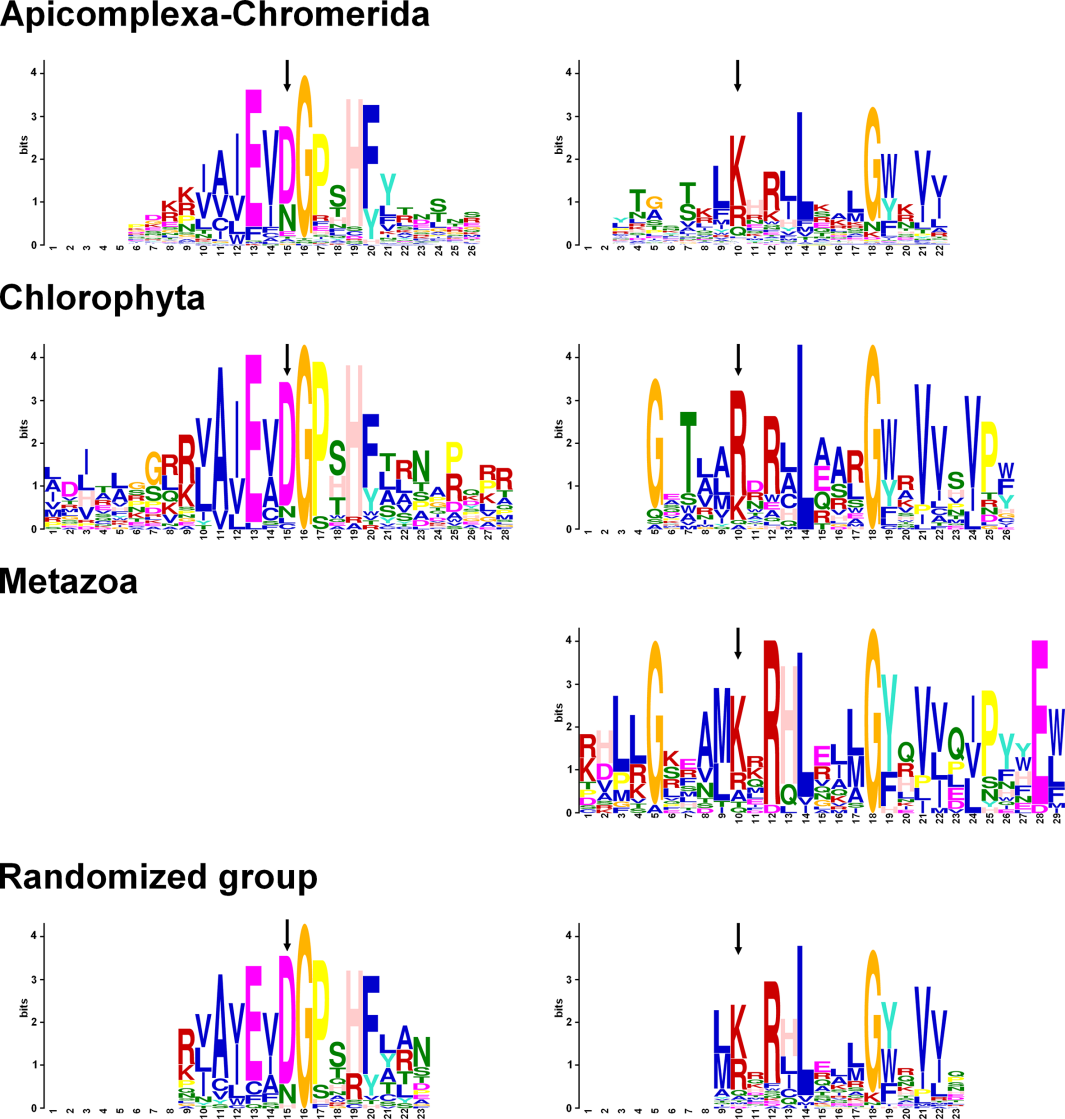
Conserved motifs identified in RAP proteins by MEME Suite. The sequences of RAP proteins from three groups, Apicomplexa–Chromerida, Chlorophyta and Metazoa, were analysed by MEME Suite. An additional group was made and regrouped 20 random sequences from each previous group. The motifs shown above are located in the RAP domain only and are aligned between them. The complete data are depicted in Fig. S5. The arrows point to the two residues of the PD-(D/E)XK endonuclease superfamily.

We then aligned the motifs identified from the RAP domain from the different groups (Fig. 4). We noticed that the first block was well conserved between Apicomplexa–Chromerida and Chlorophyta. We were able to extract a consensus sequence: NpNpNpAcNpAcGPxHF, with Np for non-polar and Ac for acidic amino acids. A second motif was detected in all of the four groups and is composed of critical residues such as a L14 and G18, with a few other amino acids enriched in R12, V21 and V22. We can assume that these residues are critical for the function of the RAP domain and that mutation of some of these residues will most likely alter the function of the protein.

Previously, RAP proteins from Metazoa and plants were associated with the repertoire of proteins belonging to the PD-(D/E)XK phosphodiesterase superfamily [12, 61]. These nucleases play diverse roles including DNA recombination and repair, tRNA splicing, and nucleic binding [61]. They shared a common core structure with αβββαβ topology, scaffolding of the conserved catalytic site, (P)DXn(D/E)XK (X is any amino acid). Further analysis of the RAP domains indicated that the residues D, D/E and K are, respectively, conserved at 53.9, 50.9 and 49 % among the 267 RAP proteins that we aligned. The aspartate/glutamate residue was identified in the Api2, Chl4 and Ran1 motifs, while the lysine was present in Ap1, Chl5, Met1 and Ran2 motifs (Figs 4 and S5). Even if these residues were globally enriched at their respective position, some RAP domains lack these amino acids, as demonstrated previously in Metazoa [12]. It is, however, important to note that different variants of this PD-(D/E)XK motif exist, which makes it difficult to identify the catalytic site on the sole basis of sequence comparisons.

As a whole, our results confirm that the RAP domain is present in evolutionarily diverse organisms and encodes conserved sequence motifs. The N-terminal region of the RAP proteins does not have an identifiably conserved motif apart from the degenerated HPR/OPR motif identified in Ran3.

### Structure predictions for RAP proteins from the apicomplexan parasite *Plasmodium falciparum*

Our data indicate that the overall structure of RAP proteins is quite conserved, suggesting its importance for their cellular function. To verify the structure of RAP proteins, we first performed HHpred searches and identified F-ATP synthase from *Polytomella* sp*.* Pringsheim 198.80 (PDB code: 6rd6 chain 2) and ribosomal molecule mL104 from *Tetrahymena thermophila* (strain SB210) (PDB code: 6z1p chain AS) as the closest modelling templates for 17 out of 22 full-length RAP proteins from *Plasmodium falciparum*. This result suggest that they have the same overall architecture consisting of helical repeats followed by the RAP domain.

**Fig. 5.**
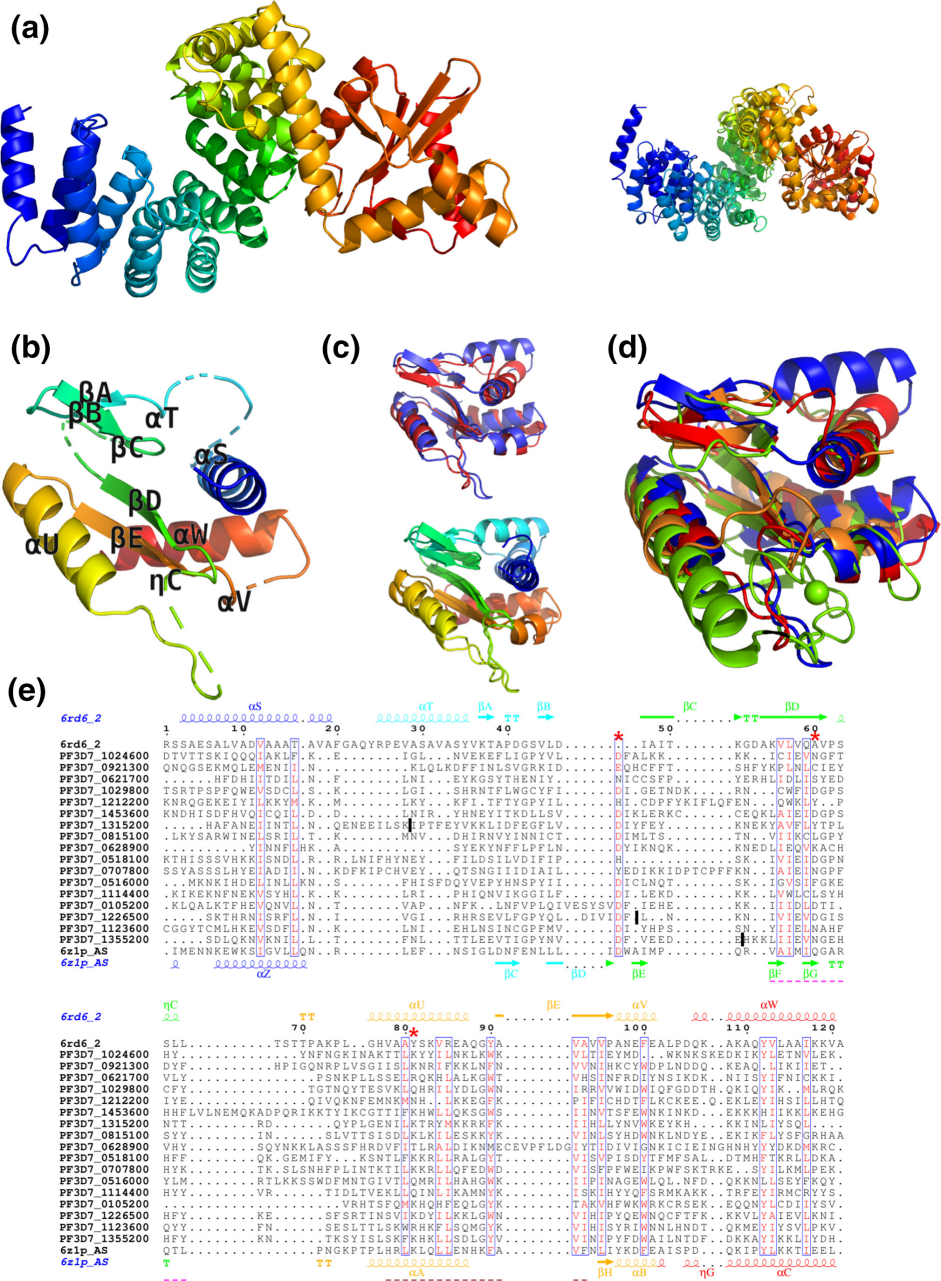
Structure predictions for RAP proteins from *Plasmodium falciparum*. (a) Left: the modelling template covering both HPRs and RAP domain from *Plasmodium falciparum* proteins, based on experimental structure of F-ATP synthase from *Polytomella* sp*.* Pringsheim 198.80 (PDB code: 6rd6 chain 2, residues 8–445). Right: the superposition of the two available modelling templates covering these domains (6rd6 chain 2 and 6z1p chain AS). (b) Structural model of the RAP domain of PF3D7_1024600 (residues 327–431), based on 6rd6 chain 2. (c) Top: the structural superposition of the two closest modelling templates for the RAP domain from PF3D7_1024600 6rd6 chain 2 residues 322–445 (blue) and 6z1p chain AS residues 591–685 (red). Bottom: the same superposition is shown in rainbow N-to-C terminus colouring. (d) Structural superposition of different modelling templates used for the RAP domain: 6rd6 chain 2 residues 322–445 (blue), 6z1p chain AS residues 591–685 (red), 3r3p chain A (orange) and 1vsr chain A (green). (**e**) Alignment of RAP domains from *Plasmodium falciparum* and F-ATP synthase (6rd6_2). The sequences and secondary structure from the two closest modelling templates are shown in the top and bottom rows of the alignment. The most conserved parts of the first and the second sequence motifs identified in Apicomplexan RAP domains (see Fig. 4, top row) are underlined with pink and magenta dashed lines, respectively. The red asterisks indicate the three residues of the PD-(D/E)XK superfamily. Positions of removed repeats are marked by dark vertical bars.

*Plasmodium falciparum* proteins are known to often contain inserts of asparagine-rich-repeats [62]. We observed such repeats in many positions inside helical repeats and RAP domains (Fig. 5a, left). The asparagine-rich regions were not aligned with modelling templates, corresponding as gaps in the template’s sequence, confirming the fact that they are not part of the conserved structural scaffold. In addition to the disruptions introduced into the alignment by these inserts, repeat domains are usually difficult to unambiguously align and model in general. Therefore, we decided not to build 3D models of the N-terminal repeats region of RAP proteins, but only test how consistent they are by structurally superimposing them. The superposition suggests that despite different biological functions and evolutionary distance between the templates, their overall structure, including arrangement of peptide repeats and RAP domains, is relatively well conserved (C⍺ RMSD (root-mean-square deviation) of 5.32 Å over 396 aligned residues, *P* value 9.24×10^−10^) suggesting that it may also be conserved in RAP proteins (Fig. 5a, right).

In the next step, we built a multiple sequence alignment and the structural model of *Plasmodium* RAP domain, but only after excising the longest asparagine-rich repeat regions from three sequences, PF3D7_1315200, PF3D7_1355200 and PF3D7_1226500 (positions of removed repeats are marked by dark vertical bars in Fig. 5e). HHpred searches identified the fragments of the same structures as for full-length proteins as the optimal modelling templates *Plasmodium* RAP domains (6rd6 chain 2 residues 322–445 and 6z1p chain AS residues 591–685). These modelling templates are highly structurally similar (C⍺ RMSD of 3.04 Å over 103 aligned residues – Fig. 5c). We selected *Plasmodium* protein PF3D7_1024600, which showed the highest similarity to the template 6rd6_2, as the representative to build a 3D model. In agreement with earlier studies [5, 12], it shows that the RAP domain has an ⍺/β-sandwich structure of a restriction endonuclease-like fold (Fig. 5b). The templates also show high structural similarity and have the same fold as modelling templates used in an earlier study of RAP domains such as 1vsr chain A and 3r3p chain A (Fig. 5c, d) [12]. The structural features of the two closest templates, 6rd6_2 and 6z1p_AS, also mostly align in the sequence alignment obtained with muscle (Fig. 5e) and, at the same time, differences between them indicate ambiguous and less accurate regions of the model. In general, the N-terminal half of the RAP domain is less conserved that its C-terminal part where the two conserved motifs identified by MEME Suite are located. Even the exact N-terminal boundary of the RAP domain could only be established by mapping the boundary of the ⍺/β domain in the template 6rd6_2 onto *Plasmodium* proteins using alignment obtained with HHpred. The most important local discrepancy between the modelling templates is the helix labelled ⍺T, which is present only in the template 6rd6_2 and appears to be present in only some of the *Plasmodium* RAP domains (Fig. 5e). Altogether, we conclude that RAP proteins share a similar overall structure, and that the RAP domain is conserved and exhibits a restriction endonuclease-like folding.

### Comparative analysis of the RAP repertoire in *Plasmodium falciparum* and *Toxoplasma gondii*

Our results described above showed that the RAP domain is abundant in the superphylum Alveolata including Apicomplexa. Even if the N-terminal regions seem to confer a certain specificity to each RAP proteins, the conservation of the structure and the RAP domain could have caused some redundancy between proteins, especially in species with a high number of RAP proteins. Thus, we selected all RAP proteins from the proteomes *Plasmodium falciparum* and *Toxoplasma gondii*, two parasites relevant to human health, in order to investigate their characteristics and potential complementarity.

To begin, we showed that *Plasmodium falciparum* has 22 RAP proteins whose 21 homologues are present in *Plasmodium berghei*, confirming the robustness of our study. In regard to *Toxoplasma gondii*, we identified 23 RAP proteins but only 12 were annotated as RAP domain-containing proteins in ToxoDB and, as indicated above, they share a low identity with *Plasmodium falciparum* proteins with the exception of TGME49_260790, which seems to be conserved across all alveolates (Fig. S2, Table S2).

To confirm the preponderance of the domain in parasite survival and to cover all predicted RAP proteins, we exploited the large-scale genetic screening methodologies to systematically identify essential genes in the respective *Plasmodium falciparum*, *Plasmodium berghei* and *Toxoplasma gondii*. In both *Plasmodium*, the essentiality was demonstrated in 34/35 RAPs in at least one of the two organisms [3, 63], while 22 out 23 RAP proteins from the *Toxoplasma gondii* genome were also described as crucial for parasite survival [2]. Altogether, 56/58 (97 %) of the tested RAP proteins were described as essential, validating not only the importance of this domain but also a clear absence of complementation between these proteins.

Another characteristic of RAP is their subcellular localization in organelles such as in the mitochondria [6, 7] or in the chloroplasts [8–10]. *Cryptosporidium* corroborates this feature since it is the only Apicomplexa that has neither normal mitochondrion and apicoplast, nor RAP proteins. We applied five subcellular localization prediction algorithms to all detected RAP proteins in *Plasmodium falciparum*, *Plasmodium berghei* and *Toxoplasma gondii* – PlasmoAP and pats for potential localization in the apicoplast, and TargetP 1.1, MitoFates and MitoProt II for potential localization in the mitochondrion (Table S2). Our analysis predicts that 18 out of 24 RAP proteins from both malaria parasites target an organelle, with 15 to the mitochondrion and 3 to the apicoplast. A similar result was obtained with *Toxoplasma gondii* with 17 RAP proteins predicted to target the mitochondria. None of the RAP proteins has been predicted to be located in the apicoplast for this parasite, but this can be explained by the fact that PlasmoAP and pats were designed for *Plasmodium* and not for *Toxoplasma*. Several experimental validations have also confirmed the localization of some of these proteins in mitochondrion or apicoplast (Table S2) [21, 53, 64, 65].

## Discussion

An important part of eukaryotic proteome is dedicated to RNA processing and metabolism. Exploring these pathways in an extensive manner will be essential to improve our understanding of gene regulation in eukaryotes. Previous studies have determined that RAP proteins are abundant in Apicomplexa [5, 54] and Dinoflagellata [53]. Here, we demonstrated that the RAP domain is present in Eukaryota, as well as a small number of bacterial and archaeal proteomes. Analysis of the bacterial RAP domains showed that the critical residues are conserved, suggesting that their detection does not seem to be false positive. Horizontal gene transfers have been proposed to explain the existence of some of the PPR proteins in bacteria, which are all symbionts or pathogens of a eukaryotic host [26, 32]. While the RAP domain detected in Archaea and Bacteria does not seem to be linked to a parasitism phenomenon, the overall depletion of RAP protein in most of these organisms suggests that several horizontal gene transfers may have taken place in distinct phyla. Among eukaryotes, some taxonomic groups or phyla, such as Amoebozoa, Nematoda, Ascomycota and Excavata, seem to be depleted in RAP proteins. We suspect that this lack of RAP proteins is most likely due to a loss during evolution. Increasing the number of organisms used in such analysis could improve our understanding of the evolution of this protein family in these particular phyla. Despite the low number of various and distant organisms analysed in this study, we detected an expansion of this protein family not only in Apicomplexa as previously described, but also in Alveolata and in Chlorophyta. The relation/dependence of these two events is, however, difficult to confirm. After years of debate on the emergence of the apicoplast in Apicomplexa, the discovery of the phylum Chromerida suggested that the apicoplast may have been the remnant of an engulfed red algae and not a green algae [66, 67]. Successive tertiary and quaternary endosymbiosis are also still considered to explain their evolution [68, 69]. Considering the role of the RAP proteins in chloroplasts and their complete absence in Rhodophyta, a single expansion of this domain cannot only be explained by a secondary endosymbiosis. No RAP proteins were detected in Glaucophyta or Rhodophyta, and only one in Streptophyta species, indicating that the expansion observed in green algae, related or not to alveolates, appeared after their divergence with the other plants. Interestingly, the HPR, identified in some of the RAP proteins, has a distribution similar, with an expansion in Alveolata (Ciliophora excluded) and the green algae, *Chlamydomonas reinhardtii* [21]. A very close link between these two families, especially given their role in the RNA metabolism of specific organelles, as well as a potential common evolution, can then be envisaged. However, it is important to note that only a fraction of the HPR/OPR proteins exhibit a classical RAP domain. Among the 22 and 25 HPR/OPR proteins in *Plasmodium falciparum* and *Toxoplasma gondii*, respectively, only 6 of them have a distinguishable RAP domain (~25 %). Overlapping between RAP and HPR/OPR proteins is even lower in Chromerida (~10–19 %). A better characterization of their respective consensus sequences could facilitate the understanding of these families and indicate how closely they are related.

Overall, RAP proteins have a common architecture with the RAP domain situated at the C-terminal region, while the N-terminal region presents helical repeats [21]. Our phylogenetic analysis demonstrated that the RAP domain is well conserved across all organisms selected, despite the evolutionary distance between them. Deep structural analysis of RAP proteins from *Plasmodium falciparum* validated this conserved architecture, strongly suggesting its essentiality for the function of these proteins. The search for conserved motifs within the domain validated a partial protection of the critical residues of the PD-(D/E)XK nuclease superfamily, although other amino acids, with an unknown role, appear to be more conserved [e.g. L14 of the second consensus motif (Fig. 4)]. To complement our analysis and validate the evolution of these proteins, we considered building a phylogenetic tree based on the N-terminal regions. Unfortunately, our initial alignments were extremely poor. This was most likely due to the very high variability of these N-terminal regions, making this analysis almost impossible. However, although the poor quality of the alignments obtained with the full-length proteins were challenging to interpret and created a significant obstacle, they showed that Aconoidasida and Chlorophyta form two distinct clades, unlike the other species studied. A RAP protein also appears to have an atypically high conservation in alveolates, which may require further research. The use of smart and MEME Suite confirmed the absence of well conserved domains/motifs in distant phyla with the exception of the HPR/OPR repeat (Ran3) motif. The FAST_1 and FAST_2 motifs were detected almost only in Metazoa, a result not entirely surprising since the structure FAST_1-FAST_2-RAP seems to have emerged early in metazoan evolution [15]. These N-terminal motifs must have conferred to these proteins specific RNA targeting, since it has indeed been demonstrated that a mutation in these regions affects the recognition of their RNA targets [12, 16]. Altogether, these results may explain the lack of complementarity observed in organisms such as in *Plasmodium* and *Toxoplasma*, despite a significant expansion of the RAP proteins [2, 3, 63].

Finally, additional studies reported the specific localization of some of these RAP proteins in plants and humans [8–10, 15, 64]. In this study, we showed that most of the RAP proteins identified in apicomplexan parasites are predicted to be located to the mitochondria or the apicoplast. The expansion of RAP proteins in Alveolata and Chlorophyta could also be linked to the presence of two distinct organelles. Although *Plasmodium* and the other apicomplexan parasites exhibit one of the smallest mitochondrial genomes with only three protein-encoding genes [70], the importance of this metabolic pathway could justify the involvement of so many regulators. It is also important to underline that the mitochondrial rRNA genes in these parasites are highly fragmented and might require the involvement of the RAP proteins to be fully functional [70–72]. Even with less complexity, this fragmentation is also found in chromerids [73], dinoflagellates [74–78] and green algae [79–81]. Such a feature has not been identified in ciliates with only a weak split of the large and small ribosomal RNA subunits [82–85], and could explain the lower number of RAP proteins observed in this phylum.

Our results broaden our understanding of the evolution of this RAP family and provide a framework for further functional investigation of these abundant proteins in apicomplexan parasites. As essential and specific to their organelles, they could be perfect targets for novel therapeutic strategies.

## Supplementary Data

Supplementary material 1

Supplementary material 2
