# Supplementary material for: Identification and phylogenetic analysis of RNA binding domain abundant in apicomplexans or RAP proteins
Source: Microb Genom. 2021 Mar 3;7(3):mgen000541. doi: 10.1099/mgen.0.000541 (PMC8190603; doi:10.1099/mgen.0.000541)
Supplement: Supplementary material 1 [file mgen-7-0541-s001.pdf]

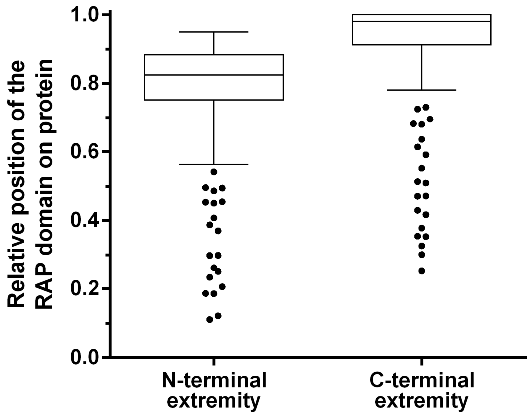

Figure S1. Relative position of the RAP domain. Tukey's box and whiskers show the positioning of the N-terminal and C-terminal ends of the RAP domains on their respective proteins. The relative values indicate N-terminal (0) and the C-terminal (1) of the full-length proteins. Proteins below 200 amino acids have been excluded (7/267).

A

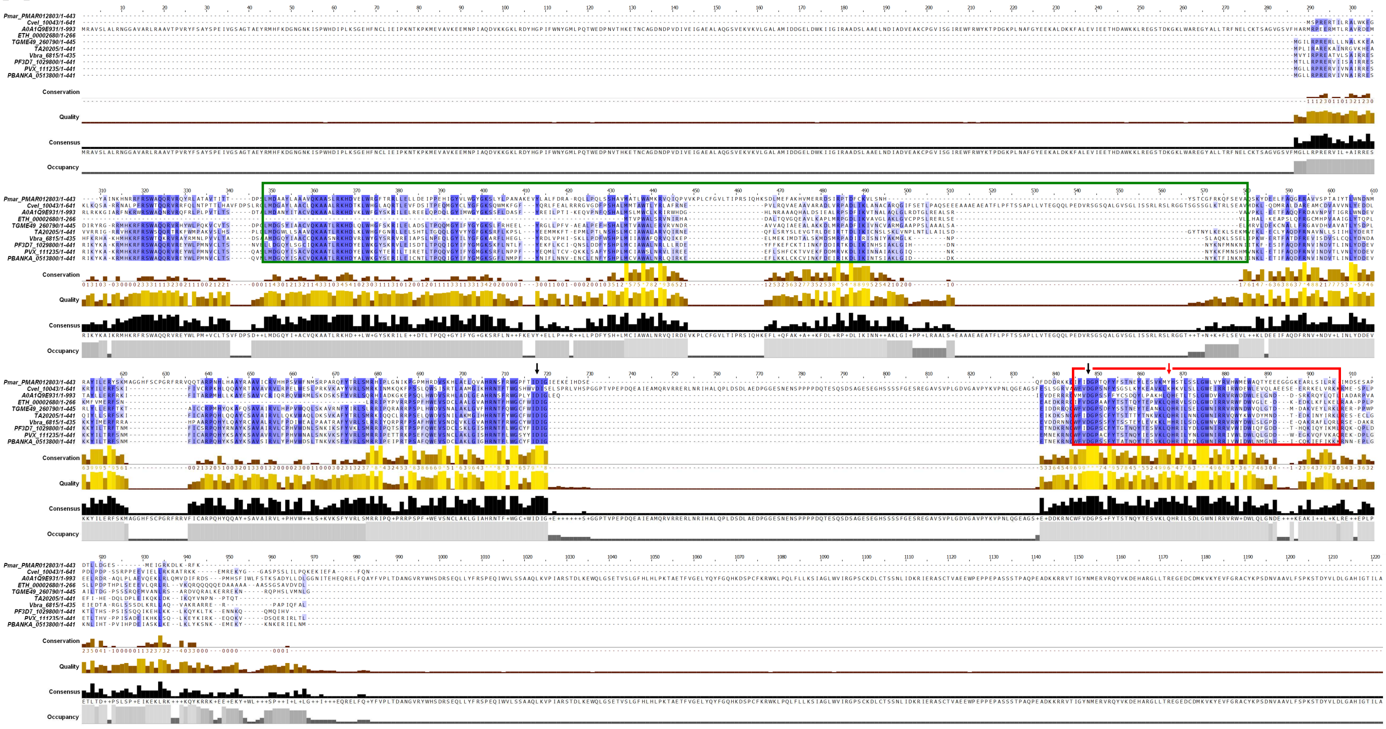

B

	P. vivax (PVX_111235)	*P. berghei* (PBANKA_0513800)	*T. annulata* (TA20205)	*T. gondii* (TGME49_260790)	*E. tenella* (ETH_00002680)	*C. velia* (Cvel_10043)	*V. brassicaformis* (Vbra_6815)	*P. marinus* (Pmar_PMAR012803)	*S. microadriaticum* (A0A1Q9E931)
P. falciparum (PF3D7_1029800)	73%	76%	49%	48%	26%	27%	47%	32%	19%

Figure S2. Global alignment of PF3D7_1029800 and its homologs. A. Global alignment between PF3D7_1029800 and its homologs from *P. vivax*, *P. berghei*, *T. annulata*, *T. gondii*, *E. tenella*, *C. velia*, *V. brassicaformis*, *P. marinus*, and *S. microadriaticum*. The positions of the peptide repeats (green box) and the RAP domain (red box) are indicated according to the respective predictions of [21] and hmmscan (Table S1). The black arrows point to the two residues of the PD-(D/E)XK endonuclease superfamily while the red arrow indicates the predicted position of the residue K, not conserved for these RAP proteins. B. Percentage of identity between PF3D7_1029800 and its homologs in the different species.

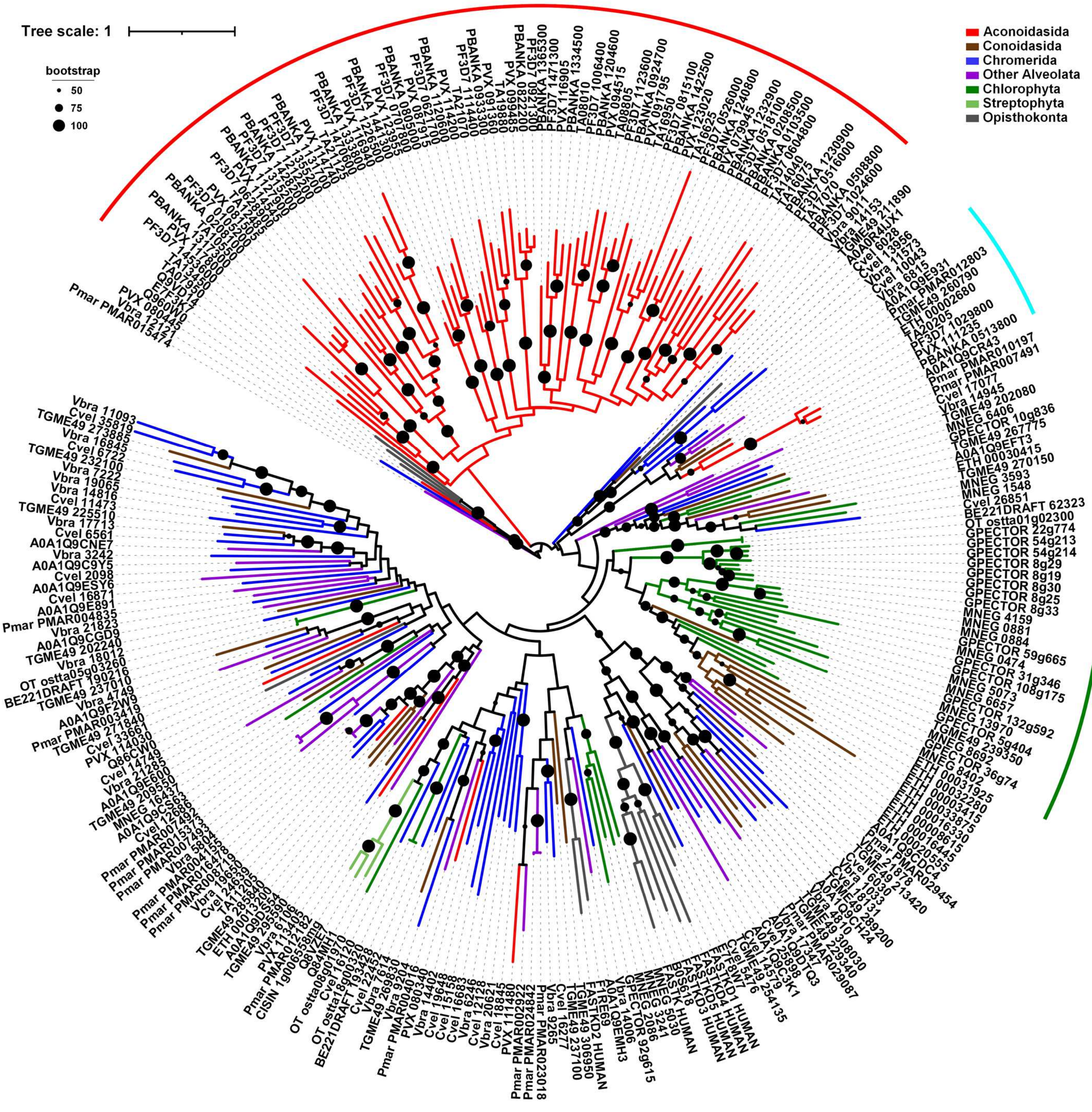

Figure S3. Phylogenetic analysis of full-length RAP proteins in Eukaryota. The maximum likelihood tree is built from 267 protein sequences corresponding to 19 different species. Alveolates are represented by Aconoidasida (*P. falciparum*, *P. vivax*, *P. berghei*, and *T. annulata*) in red, Conoidasida (*T. gondii* and *E. tenella*) in brown, Chromerida (*V. brassicaformis* and *C.velia*) in blue and *P. marinus* and *S. microadriaticum* in purple. Viridiplantae are represented by Chlorophyta (*G. pectorale*, *M. neglectum*, and *O. tauri*) in green and Streptophyta (*A. thaliana*, *O. sativa*, and *C. sinensis*) in pale green. Opisthokonta (*H. sapiens*, *D. rerio*, and *D. melanogaster*) are indicated in grey. Bootstrap values (> 50%) are shown on respective branches.

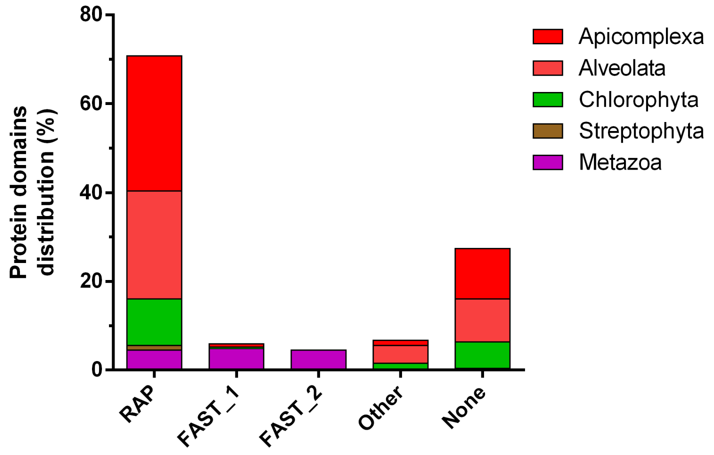

Figure S4. Domains identified in RAP proteins by SMART. The complete set of full-length proteins selected in this study (267 sequences) were analyzed by SMART. The results obtained for each protein are available in Table S1.

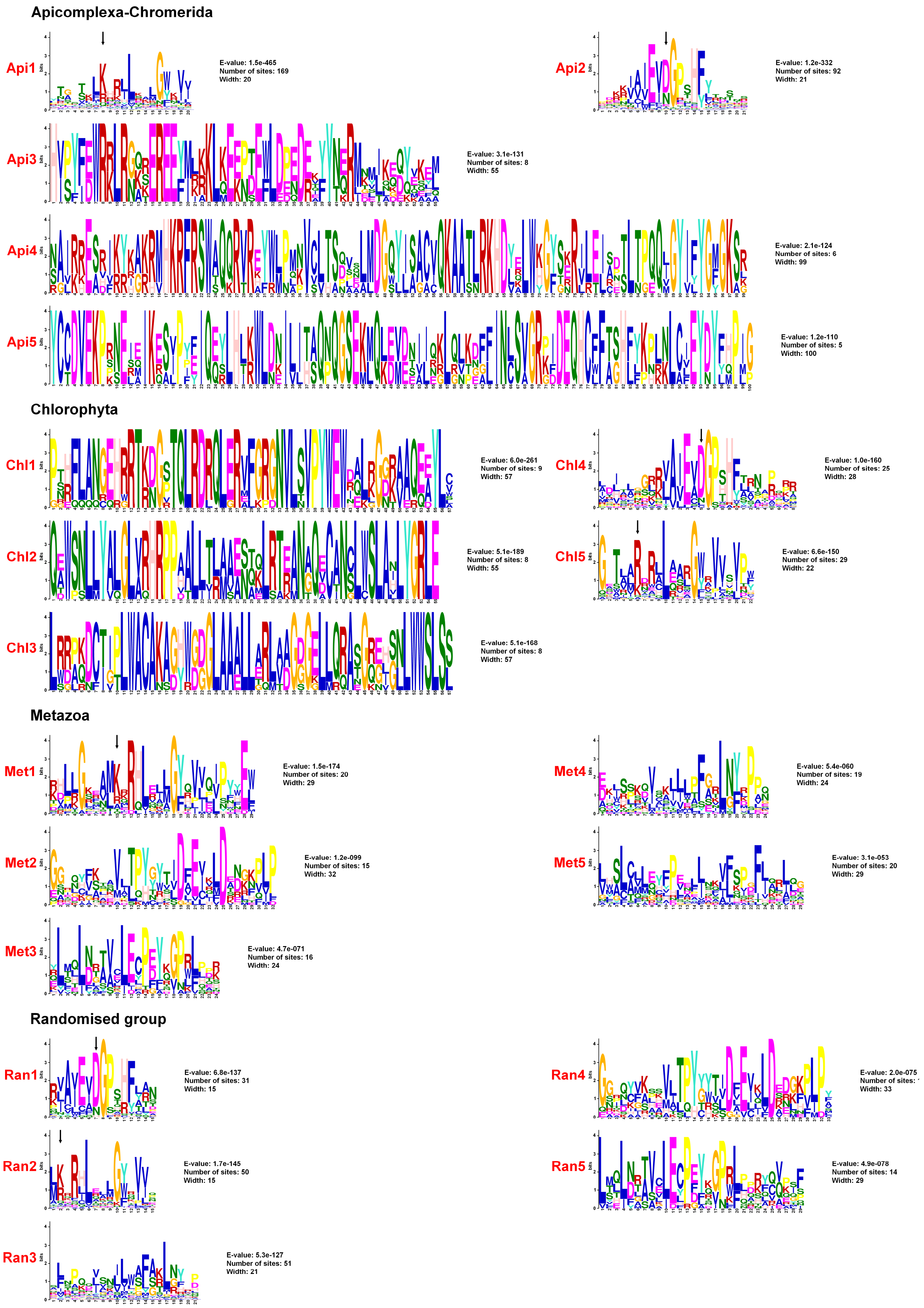

Apicomplexa-Chromerida
Api1
E-value: 1.5e-465
Number of sites: 169
Width: 20
Api2
E-value: 1.2e-332
Number of sites: 92
Width: 21
Api3
E-value: 3.1e-131
Number of sites: 8
Width: 55
Api4
E-value: 2.1e-124
Number of sites: 6
Width: 99
Api5
E-value: 1.2e-110
Number of sites: 5
Width: 100
Chlorophyta
Chl1
E-value: 6.0e-261
Number of sites: 9
Width: 57
Chl4
E-value: 1.0e-160
Number of sites: 25
Width: 28
Chl2
E-value: 5.1e-189
Number of sites: 8
Width: 55
Chl5
E-value: 6.6e-150
Number of sites: 29
Width: 22
Chl3
E-value: 5.1e-168
Number of sites: 8
Width: 57
Metazoa
Met1
E-value: 1.5e-174
Number of sites: 20
Width: 29
Met4
E-value: 5.4e-060
Number of sites: 19
Width: 24
Met2
E-value: 1.2e-099
Number of sites: 15
Width: 32
Met5
E-value: 3.1e-053
Number of sites: 20
Width: 29
Met3
E-value: 4.7e-071
Number of sites: 16
Width: 24
Randomised group
Ran1
E-value: 6.8e-137
Number of sites: 31
Width: 15
Ran4
E-value: 2.0e-075
Number of sites:
Width: 33
Ran2
E-value: 1.7e-145
Number of sites: 50
Width: 15
Ran5
E-value: 4.9e-078
Number of sites: 14
Width: 29
Ran3
E-value: 5.3e-127
Number of sites: 51
Width: 21

Figure S5. Motifs identified in RAP proteins by MEME Suite. The sequences of RAP proteins from three groups, Apicomplexa-Chromerida, Chlorophyta and Metazoa, were analyzed by MEME Suite. An additional group was made and regrouped 20 random sequences from each previous group. The e-values, number of sites detected and the sequence width are indicated. The arrows point to the two residues of the PD-(D/E)XK endonuclease superfamily.
